# Vulvovaginal Infralevator Haematoma Mimicking the Second Stage of Labour

**DOI:** 10.1155/2017/8062793

**Published:** 2017-01-18

**Authors:** J. O. Awoleke, O. M. Ipinnimo

**Affiliations:** ^1^Department of Obstetrics and Gynaecology, Ekiti State University, Ado Ekiti, Ekiti State, Nigeria; ^2^Department of Obstetrics and Gynaecology, Ekiti State University Teaching Hospital, Ado Ekiti, Ekiti State, Nigeria

## Abstract

Even though they are quite uncommon, puerperal genital haematomas can be associated with serious maternal morbidity. Key findings are significant perineal pain and, depending on the location, visible swelling. However, attention can be drawn to its progression by the rare occurrence of persistent painful “bearing down” efforts, even after the successful delivery of the baby. The final size of this haematoma and the rare presentation make it truly uncommon. The primary goals of treatment include the prevention of further blood loss, minimizing tissue damage, relieving pain, and reducing the risk of infection. Management is generally conservative for small collections, but surgery is indispensable when they acutely expand in size or are large with worsening symptoms.

## 1. Introduction

Vulvovaginal haematoma (VVH) occurring postpartum is a relatively uncommon complication of labour but can be a cause of serious morbidity and even maternal death [1]. This is because the symptoms could be nonspecific and bleeding is usually concealed, leading to diagnostic difficulties and delayed treatment.

Clinically significant haematoma requiring surgical intervention occurs in about 1 in 1000 vaginal deliveries, while estimates from case series report incidences of 1 : 500 to 1 : 12,500 vaginal births [2]. Frequently cited risk factors include nulliparity, prolonged second stage of labour, instrumental delivery, poorly repaired episiotomy and lacerations, delivery of a baby > 4 kg, varicosities of the genital tract, and maternal age > 29 years [3].

Vulvovaginal haematomas classically present a few hours after delivery. The patient is usually restless, complaining of pelvic pain, inability to pass urine or rectal tenesmus. The rare presentation with the urge to “bear down” may confuse the accoucheur who is not conversant with this pathology leading to the possibility of extensive tissue damage and postpartum collapse from hypovolemic shock before an accurate diagnosis is made [4].

We therefore present the case of a nullipara with puerperal VVH that mimicked the “bearing down” efforts of the second stage of labour.

## 2. Case Presentation

A 26-year-old now Para 1 was referred from a peripheral hospital to the Teaching Hospital because of persistent “bearing down” efforts after a spontaneous vaginal delivery of a live male neonate with birth weight of 3400 gm six (6) hours prior to presentation. The labour had lasted about 9 hours. The birth attendant, having examined the patient again and excluded the possibility of an undiagnosed twin, decided to refer her for expert management.

Examination on admission revealed a young lady who was pale, dehydrated, and intermittently straining in response to “the irresistible urge to push.” Her pulse rate was 102 beats/min, regular and of moderate volume with a blood pressure of 120/70 mmHg. The uterus was about 20-week pregnancy size and was well contracted. Pelvic examination revealed a blood-smeared vulva with a tender swelling involving the right labia minus and majus measuring about 4 cm by 4 cm.

**Figure 1:**
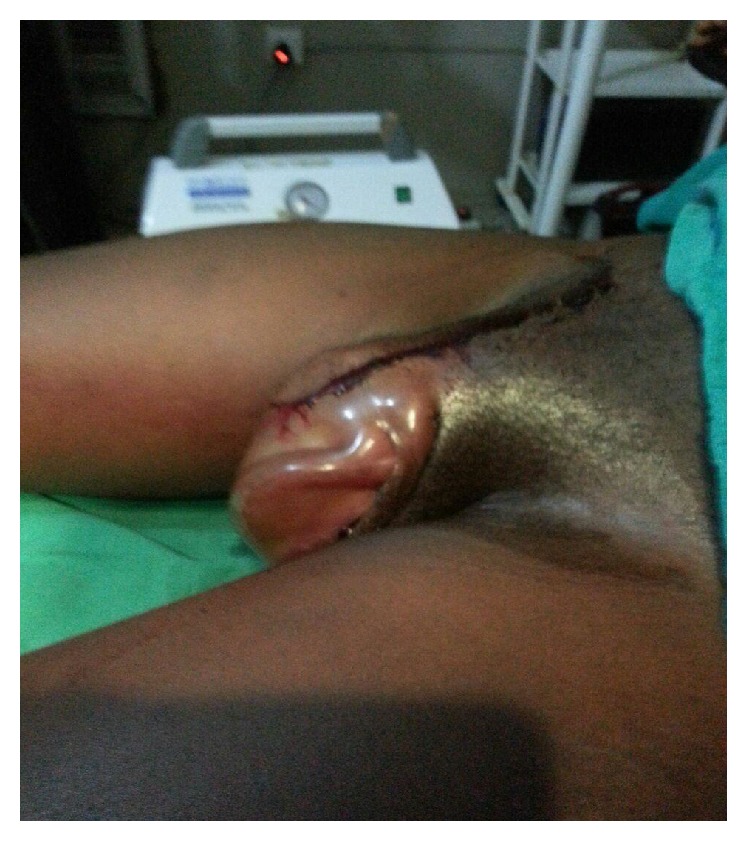
Preoperative VVH.

**Figure 2:**
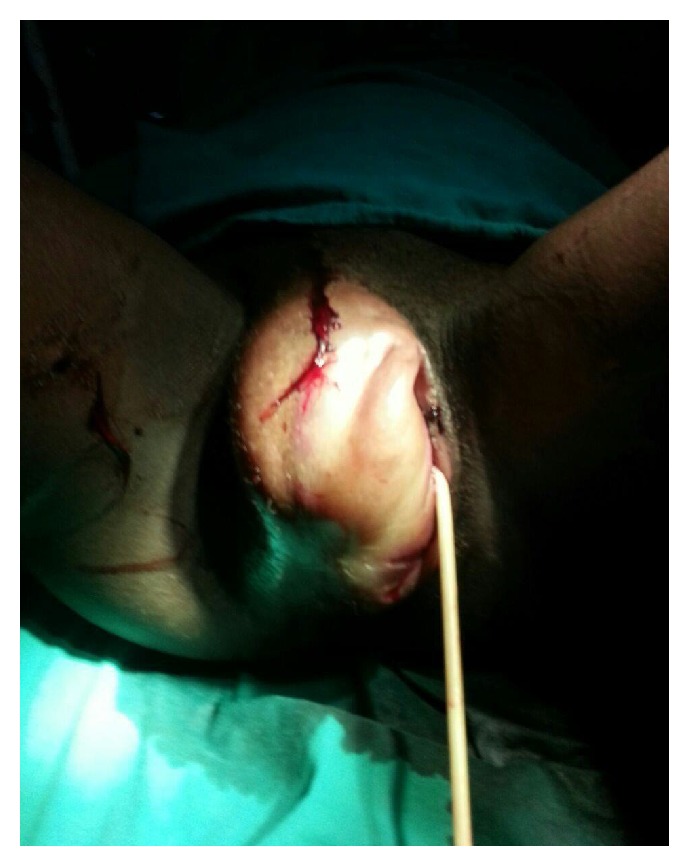
The VVH viewed with patient in the dorsal position (urethral catheter in situ).

A diagnosis of puerperal genital haematoma was made. The plan was to institute conservative management with vaginal pack and analgesics. Intravenous access was established and blood sample was obtained for packed cell volume (PCV) (which was 22%) and grouping and cross-matching of three units of whole blood. The limits of the swelling were ink-marked and observed for one hour to confirm if there would be an enlargement. Progressive expansion of the VVH occurred, necessitating surgical intervention. The intraoperative findings included a huge 12 cm by 10 cm right vulvovaginal haematoma completely occluding the vaginal cavity and stretching the overlying perineal skin (Figures 1 and 2).

**Figure 3:**
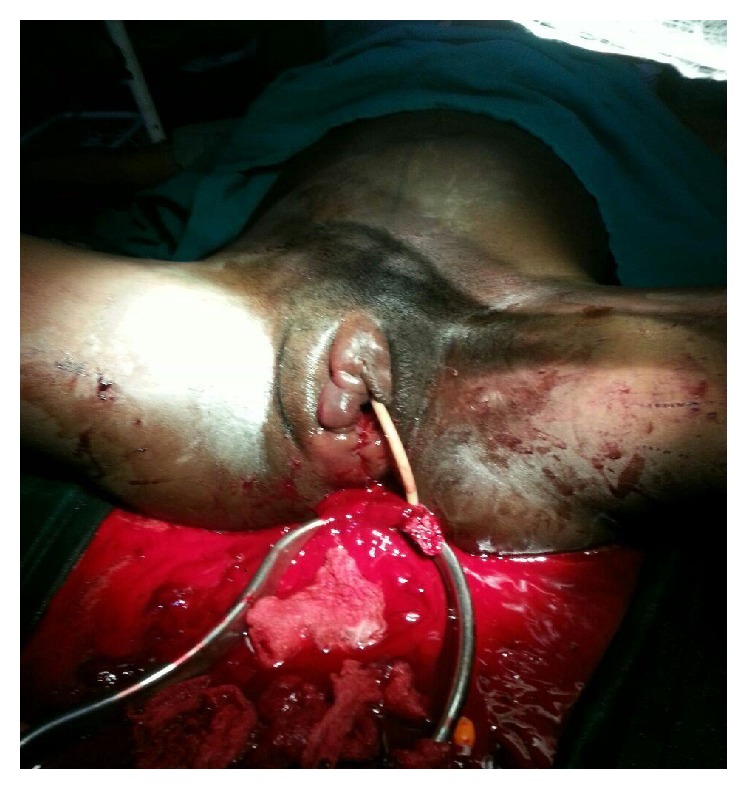
Evacuated clots, closed dead space.

There was no significant bleeding from the vagina. Incision and evacuation of the haematoma and exploration of the cavity were done and 1500 mls of clotted blood was evacuated from the cavity. Haemostasis was achieved with “figure-of-eight” sutures. The space was obliterated with deep sutures and the overlying skin approximated carefully, avoiding injury to the urethra. The vagina was packed with gauze to further assist with haemostasis (Figure 3). She had broad-spectrum antibiotics and transfusion of three units of blood and was discharged home on the third day following a satisfactory postoperative recovery.

## 3. Discussion

Puerperal VVH are rare and have not been extensively reported. They are usually unilateral and in most cases develop insidiously, attention being drawn to them when the woman collapses in shock, groans in pain, or complains of “bearing down” pain* after* vaginal delivery [4]. This is usually due to rupture of the labial branches of the ipsilateral internal pudendal artery [5], which can occur without laceration of the superficial tissue. In about 20% of the cases, radial stretching of the birth canal occurs as the fetus passes through leading to rupture of the blood vessels [6]. Since the subcutaneous tissue in the vagina is quite pliable, haematomas from the concealed bleeding can achieve massive dimensions before expansion ceases [7]. The cases present with a huge vulval swelling that is associated with pain and may progress to occlude the vaginal orifice causing urinary retention and hypovolemic shock. The persistent involuntary urge in this patient to bear down when the VVH was still quite small makes this case a true rarity.

Management options are either conservative or surgical depending on the size and rate of progression of the haematoma. Conservative management was initially instituted in the patient presented because the swelling was less than 5 cm. Other conservative measures employed when the haematoma is <5 cm and static/not rapidly increasing in size include ice packs, pressure dressing, bladder drainage, and analgesics [8]. The visible skin margin of the haematoma could also be marked to establish progression. However, in swellings > 5 cm, the conservative approach was associated with longer hospital stay, increased need for antibiotics and blood transfusion, and greater recourse to surgical intervention [7].

Following evidence of progression or in large haematomas, surgical management should be instituted with the incision made as medially as possible to minimize visible scarring [1]. The presence of any residual bleeding into the haematoma cavity (which was not present in this case) is an indication for the insertion of a drain, which should be left in place for at least 12–24 hours [9]. The repair should be done in such a way as to obliterate the dead space to prevent recollection and abscess formation.

Continuing bleeding should be excluded by close monitoring of the operation site, evaluation of the vital signs, and urinary output. When it occurs, the site should be reexplored and identified bleeders ligated. Sometimes, ligation of the internal iliac artery or even hysterectomy may be necessary [1] or use of angiographic embolization for cases of on-going bleeding not controlled by conventional surgical techniques [10].

To prevent infection, broad-spectrum antibiotics should be given. Good analgesia and close observation are important postoperatively. Prompt resolution of haematoma will improve outcome and result in reduced scarring, postpartum pain, and dyspareunia [11].
